# Development of a population pharmacokinetic model of prucalopride in children with functional constipation

**DOI:** 10.1002/prp2.236

**Published:** 2016-06-01

**Authors:** Erno van Schaick, Marc A. Benninga, Amy Levine, Mats Magnusson, Steven Troy

**Affiliations:** ^1^ SGS Exprimo NVMechelenBelgium; ^2^ Division of Pediatric Gastroenterology and NutritionEmma Children's Hospital/Academic Medical CenterAmsterdamNetherland; ^3^ ShireLexington, MassachusettsUSA; ^4^ Pharmetheus ABU‐A Science ParkUppsalaSweden

**Keywords:** Children, constipation, pediatric, population pharmacokinetics, prucalopride

## Abstract

A recent phase 3 trial of prucalopride in children with functional constipation (SPD555‐303 ClinicalTrials.gov Identifier: NCT01330381) reported negative efficacy results. Here, we developed a population pharmacokinetic (PK) model of prucalopride in children to assess prucalopride exposure in SPD555‐303. An initial population PK model in children was developed based on sampled single‐dose data from a phase 1 study (PRU‐USA‐12). This model was subsequently updated with sampled data from SPD555‐303 and used to simulate plasma concentration–time profiles for children aged 6 months to 18 years who were treated once daily with prucalopride 0.02, 0.04, or 0.06 mg kg^−1^ (maximum dose, 2 mg). Simulated PK profiles were compared with those of adults at the recommended dose of 2 mg once daily. Data were available from 38 patients (median age, 8.5 years) in PRU‐USA‐12 and 137 patients (median age, 7.9 years) in SPD555‐303. Mean (range) area under the plasma concentration–time curve (AUC) at steady state was 62.3 (40.5–82.7) ng mL^−1^ h (dose, 0.03 mg kg^−1^) in PRU‐USA‐12 and 100.3 (22.7–286.0) ng mL^−1^ h (dose, 0.04 mg kg^−1^; maximum, 2 mg) in SPD555‐303. Prucalopride 0.04 mg kg^−1^ once daily in children produced similar maximum plasma concentrations and approximately 10% lower AUC compared with adults receiving 2 mg once daily. This population PK analysis indicates that the PK profile of prucalopride in children in SPD555‐303 was similar to that observed in adults. The negative efficacy results of SPD555‐303 cannot be explained by differences in prucalopride exposure between children and adults.

**Abbreviations**
5‐HT_4_: 5‐hydroxytryptamine type 4
Arb: air resources board
AUC: area under the plasma concentration–time curve
CL: clearance
*C*_max_: maximum plasma concentration
*C*_min_: minimum drug concentration
CrCL: creatinine clearance
CWRES: conditional weighted residuals
FOCE: First‐Order Conditional Estimation
FWHM: full width at half‐maximum
GFR: fractional glomerular filtration rate
HESI: heated electrospray interface
HPLC: high‐performance liquid chromatography
*K*_*a*_1: one absorption rate
*K*_*a*_2: second absorption rate
LLOQ: lower limit of quantification
MTIME: estimated cut‐off time
PK: pharmacokinetics
PMA: postmenstrual age
Q: intercompartmental clearance
Q–Q: quantile–quantile
*V*2: volume of distribution of the central compartment
*V*3: volume of distribution of the peripheral compartment
WT: weight

## Introduction

Constipation is a common problem in children, with estimates of prevalence ranging from 0.7% to 29.6% (Mugie et al. 2011). Childhood constipation continues into adulthood in at least one‐quarter of cases, and, if left untreated, can lead to fecal incontinence and associated psychological problems (Bernard‐Bonnin et al. 1993). However, evidence for the effectiveness of laxative therapy in children is largely inconclusive (Pijpers et al. 2009). The 5‐hydroxytryptamine type 4 (5‐HT_4_) receptor agonist cisapride was initially approved for the treatment of constipation in children; however, this drug was subsequently withdrawn from the market owing to serious adverse effects (US Food and Drug Administration, 2000). A gastrointestinal prokinetic agent that is effective and has a good safety profile would be a beneficial therapeutic option in the management of childhood constipation.

Prucalopride is a selective, high‐affinity, 5‐HT_4_ receptor agonist that is licensed in Europe for the treatment of chronic constipation in adult men and women for whom laxatives have failed to provide adequate relief (Shire. Resolor SmPC). The pharmacokinetics (PK) of prucalopride have been described in adults, and the recommended dose in patients aged 18 years and older is 2 mg once daily, with a starting dose of 1 mg once daily in those aged over 65 years (Shire. Resolor SmPC). Several randomized clinical trials have demonstrated the efficacy of prucalopride in increasing the frequency of bowel movements and improving patient‐reported symptoms and quality of life in adults at these doses (Camilleri et al. 2008; Quigley et al. 2009; Tack et al. 2009; Ke et al. 2012; Yiannakou et al. 2015).

To date, two separate studies have been conducted to assess prucalopride in pediatric populations (Winter et al. 2013; Mugie et al. 2014). Winter et al. (2013) reported the results of a phase 1 study that assessed the efficacy, safety, and PK of prucalopride in children with functional constipation. The initial phase 1 study (PRU‐USA‐12; ClinicalTrials.gov Identifier: NCT01674166) assessed the PK and safety of a single dose of prucalopride for up to 72 h postdose in children aged 4–12 years with functional constipation. Subsequently, 37 of the 38 patients who completed this study were entered into an 8‐week, open‐label, follow‐up study assessing the efficacy and safety of prucalopride (Winter et al. 2013). The results of the first study suggested that children receiving a single dose of prucalopride 0.03 mg kg^−1^ (approximately equivalent to a 2 mg dose in an adult weighing 70 kg) had a generally similar PK profile to that observed in adults, but that the children exhibited a lower systemic clearance (expressed as L/h/kg) than adults leading to a 30–40% lower systemic exposure. Linear predictions of dose based on weight (per kg model, i.e., 2 mg dose in a 70 kg adult transforms to 0.03 mg kg^−1^) are used commonly as an initial selection of pediatric doses, but pediatric doses may need to be refined as clearance may more likely change according to the allometric 3/4 power model (Anderson and Holford 2008, 2013). Efficacy data from the follow‐up study suggested an improvement in the frequency of bowel movements and stool consistency over 8 weeks in children treated with prucalopride (Winter et al. 2013).

More recently, Mugie et al. (2014) reported the results of a randomized, placebo‐controlled, double‐blind, phase 3 trial to evaluate the efficacy and safety of prucalopride in children and adolescents (6 months to 18 years old) with functional constipation (SPD555‐303; ClinicalTrials.gov Identifier: NCT01330381). The results of this trial showed that prucalopride was well tolerated, but was no more effective than placebo in children with functional constipation. In this study, sparse blood sampling at steady state was carried out to enable the secondary objective of population PK modeling of prucalopride to be achieved.

In this analysis, we developed a population PK model in children to provide a good description of the PK of prucalopride in children and to evaluate prucalopride exposure in the SPD555‐303 study.

## Materials and Methods

### Overview of analytic approach

The population PK model presented here was developed in a stepwise manner from a population model developed in adults (data not shown). The model in adults was a two‐compartment model with a lag time followed by a first‐order absorption process, which was subsequently adapted for a pediatric population using allometric scaling on weight and adjustment of age‐related maturation of renal function. The model was further externally evaluated using the datasets from PRU‐USA‐12 and SPD555‐303 (Winter et al. 2013; Mugie et al. 2014). A visual predictive check and a comparison of observed and predicted PK parameters showed that the adapted model could predict both prucalopride's maximum concentration and its overall exposure in children. However, parameters describing the distribution into the peripheral compartment were more rapid and more pronounced than predicted from the adapted model, and were updated accordingly. These adjustments were shown to result in an accurate prediction.

The pediatric population PK model was used to simulate prucalopride exposure and to guide dose selection in a pediatric population aged 6 months to 18 years. The analysis described in this manuscript entails an empirical Bayesian prediction and a subsequent update of the model using sparsely sampled data from SPD555‐303. The validity of the final model and consistency of the data were assessed using goodness‐of‐fit plots (predicted vs. observed plasma prucalopride concentrations); evaluation of the distributions of individual parameters (quantile–quantile [Q–Q] plot of conditional weighted residuals (CWRES) vs. normal scores; CWRES vs. population predicted scores; CWRES vs. time) and evaluation of parameter shrinkage. A limited additional model optimization was conducted to evaluate potential differences between the phase 1 and phase 3 trial datasets. Finally, the updated model was used to simulate expected plasma concentration–time profiles for pediatric patients aged 6 months to 18 years when treated once daily with prucalopride 0.02, 0.04, or 0.06 mg/kg (maximum dose, 2 mg); these simulations were compared with adult profiles to determine the optimal pediatric dose of the drug. Additional details are provided below.

### Primary trial design and study population

The study design and key efficacy and safety end points of the two trials used in the analysis have been described in detail elsewhere (Winter et al. 2013; Mugie et al. 2014). Brief descriptions of the study designs are given below and an overview is provided in Table 1. Both studies were conducted in accordance with the International Conference on Harmonisation Guidelines for Good Clinical Practice (International Conference on Harmonisation of Technical Requirements for Registration of Pharmaceuticals for Human Use, 1996), the principles of the Declaration of Helsinki (World Medical Association, 2013), and local ethical and legal requirements. Written informed consent was obtained and signed by each child's legal guardian and by the investigator before the initiation of any study procedures.

**Table 1:** Designs of the two pediatric studies of prucalopride included in the population PK model

	PRU‐USA‐12	SPD555‐303
Study phase	1	3
Number of patients in safety set	38 (13 girls)	213 (118 girls)
Number of patients in PK dataset	38 (13 girls)	137 (79 girls)
Type of patients	Children aged ≥4 years to ≤12 years with functional constipation	Aged from ≥6 months to 18 years with functional constipation
Prucalopride dose	0.03 mg kg^−1^ (0.02 mg kg^−1^ administered to one patient)	Weight ≤ 50 kg: prucalopride 0.04 mg kg^−1^ body weight. After 4 weeks of treatment, this dose had to be adjusted to prucalopride 0.06 mg^ ^kg^−1^ once daily when there was insufficient response and no safety concerns. The dose had to be reduced to 0.02 mg kg^−1^ in case of safety/tolerability concerns and a sufficient response
Rescue medication	N/A	Bisacodyl 5 mg (one tablet) or sodium picosulfate droplets (7.5 mg mL^−1^, 1 droplet/5 kg body weight)
Single/multiple dose	Single	Multiple dose
Formulation	0.2 mg mL^−1^ oral solution	0.4 mg mL^−1^ oral solution and 2 mg tablet
Food	Site‐specific standardized snack of milk and cookies 2 h after dosing	Without food (1–3 h before the evening meal), except for the first dose, which might occur earlier to facilitate obtaining a 1–3‐h sample
Sampling time windows and number of samples	Sampling for 72 h postdose; mean, 12.6 samples per patient	First dose: one blood sample 1–3 h postdose; two samples at steady state at week 8 and week 24 (14–26 h postdose)
Assay (LLOQ)	RIA (0.1 ng mL^−1^)	LC‐MS/MS (0.2 ng mL^−1^)

LC‐MS/MS, liquid chromatography–tandem mass spectrometry; LLOQ, lower limit of quantification; N/A, not applicable; PK, pharmacokinetic; RIA, radioimmunoassay.

#### PRU‐USA‐12 study

Children were included in the study if they were aged 4–12 years, with Tanner stage I–II physical development (Marshall and Tanner 1969, 1970) and functional constipation (defined as a history of fecal impaction occurring periodically in the past 2 months in addition to fewer than three bowel movements per week and/or a history of fecal incontinence). They received a single dose of prucalopride 0.03 mg kg^−1^ in oral solution (0.2 mg mL^−1^) taken with 30‐mL water. Two hours after dosing, children received a site‐specific standardized snack of milk and cookies. Blood samples of 1 mL each were taken at the following times postdose: 0.5, 1, 1.5, 2, 3, 4, 6, 8, 12, 18, 24, 48, and 72 h. Urine was collected before prucalopride administration and for 0–6 h, 6–12 h, and 12–24 h periods postdose. Prucalopride concentration was determined in plasma using a validated radioimmunoassay technique described previously (Winter et al. 2013), which is linear over the range 0.1–55.0 ng mL^−1^ and has a lower limit of quantification (LLOQ) of 0.1 ng mL^−1^. Accuracy and precision were measured for the quality control samples (0.17–10.1 ng mL^−1^): the mean accuracy ranged from 90.8% to 97.7% and overall precision from 5.0% to 13.4%.

#### SPD555‐303 study

Children aged from 6 months to 18 years with functional constipation were treated with prucalopride or placebo in an 8‐week double‐blind phase, and were then entered into a 16‐week open‐label, active comparator phase (PEG 4000). Although the inclusion criteria specified an age of 6 months or older, in practice, no patients younger than 1 year were recruited. Patients were defined as having functional constipation if they had two or fewer spontaneous bowel movements per week with at least one of the following during the month (or 2 months for those aged ≥4 years) before inclusion in the study: at least one episode of fecal incontinence per week (after acquisition of toileting skills); history of retentive posturing or excessive volitional stool retention; history of painful or difficult bowel movements; presence of a large fecal mass in the rectum; or history of large‐diameter stools.

Patients were randomized to receive once‐daily doses of placebo or prucalopride 0.04 mg kg^−1^ up to a maximum dose of 2 mg. After 4 weeks of treatment, the dose could be adjusted for each patient (maximum total dose of 2 mg once daily). A dose increase to 0.06 mg kg^−1^ had to occur if there were no safety concerns and there was an insufficient response to treatment, and a dose decrease to 0.02 mg kg^−1^ had to occur if there were safety/tolerability concerns that were likely to be related to treatment and there was a sufficient response to treatment.

Sparse PK blood sampling was conducted: one sample was collected at the start of treatment on day 1 (1–3 h postdose, close to maximum plasma concentration [*C*_max_]) and two samples were collected at steady state, after 8 and 24 weeks of treatment (at 14–26 h postdose, close to expected minimum drug concentration [*C*_min_]).

Prucalopride concentrations were determined from blood samples using a validated high‐performance liquid chromatography–tandem mass spectrometry method. The LC/MS/MS system was comprised of the following devices (Shimazdu Corporation, Columbia, MD): SIL‐30ACMP autosampler set to 4°C; CBM‐20A controller; CTO‐20AC column oven set to 25°C; and LC‐30AD pumps. Mobile phase A consisted of 0.1 mol/L ammonium acetate in reverse osmosis water (pH 7.0). Mobile phase B consisted of 1 mol/L ammonium acetate in reverse osmosis water (pH 7.0): MeOH: ACN (10:45:45, v/v/v). An Xbridge C18, 4.6 × 250 mm, 5 *μ*m analytical column was employed (Waters, Milford, MA), and flow rate was set to 0.8 mL/min. The high‐performance liquid chromatography (HPLC) gradient started at 100% mobile phase A, ramped to 20% mobile phase B in 1 min; ramped from 20 to 35% mobile phase B in 9 min; ramped to 37% mobile phase B in 6 min; ramped to 100% mobile phase B in 14 min and held for 5 min, eventually returned to initial conditions in 0.1 min, and re‐equilibrated for 4.9 min.

A Q Exactive^™^ mass spectrometer with Xcalibur 2.2 software was utilized (Thermo Fisher Scientific, Waltham, MA). The following MS conditions were employed with the heated electrospray interface (HESI): positive ion or positive/negative ion switching polarity; capillary temperature set to 300°C; sheath gas flow rate was 75 arb; auxiliary gas flow rate was 15 arb; sweep gas flow rate was 3 arb; S‐lens level was 50; and heater temperature was 500°C. The survey scan event cycle range was m/z 100–1000 at resolution 70,000 full width at half‐maximum (FWHM), and the data‐dependent scans were at resolution 35,000 FWHM or 17,500 FWHM.

The method was linear over the range 0.2–100 ng mL^−1^ and had an LLOQ of 0.2 ng mL^−1^. Accuracy and precision were measured for the quality control samples (0.60–80 ng mL^−1^): the mean accuracy ranged from 99.2% to 103.2% and the overall precision from 3.9% to 4.1%.

### Population pharmacokinetic model design

**Figure 1:**
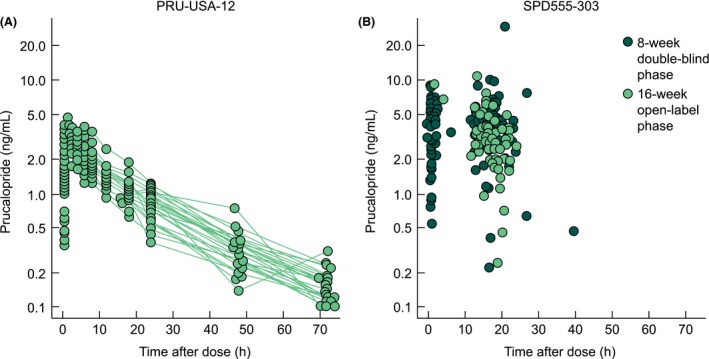
Plasma concentration–time profiles for prucalopride after (A) a single dose of 0.02–0.03 mg kg^−1^ in children aged 4–12 years in study PRU‐USA‐12 and (B) after multiple doses of prucalopride 0.02–0.06 mg kg^−1^ in children aged 1–18 years in study SPD555‐303.

All analyses were performed in accordance with the relevant industry guidelines (European Medicines Agency, 2007 and US Food and Drug Administration, 1999), using a nonlinear mixed‐effects modeling tool (NONMEM^®^ version 7.2.0; Sheiner and Beal 1980, 1981, 1983), and were processed using R version 2.15.2 (R Foundation for Statistical Computing, Vienna, Austria). Exploratory graphical analysis was carried out to evaluate the model structure. Generally, the plasma concentration–time profile followed a biexponential disposition (Fig. 1); therefore, a two‐compartment PK model was considered likely to provide an appropriate description of the sparsely sampled data.

#### Structural model components

Prucalopride concentration–time data were described by a two‐compartment model with two sequential first‐order absorption processes. In this model, one absorption rate (*K*_*a*_1) was applied before an estimated cut‐off time (MTIME) and a second absorption rate (*K*_*a*_2) applied after this cut‐off time. Interindividual variability was estimated for clearance (CL), the two absorption rate parameters (*K*_*a*_1 and *K*_*a*_2), and the central (*V*2) and peripheral (*V*3) volumes of distribution, and was described using an exponential error model. The residual variability was explained with an additive error on log‐transformed data. The absorption parameters and the relative bioavailability (85.8%, data on file) were fixed to the value previously estimated in adults. In the current analysis, the model was updated by estimating separate parameters for CL and associated interindividual variability for PRU‐USA‐12 (CL or CL_PRU‐USA‐12_) and SPD555‐303 (CL2 or CL_SPD555‐303_). Parameter estimation was performed using the First‐Order Conditional Estimation (FOCE) with interaction.

#### Allometric scaling and age‐related maturation

To enable the model to describe prucalopride PK in children of different ages and body weights, allometric scaling principles and maturation of renal function were included in the model (Anderson and Holford 2008). The sample size was too small to perform a formal step‐wise covariate search (Ribbing and Jonsson 2004), but the following patient covariates were considered in the analysis because they were expected to impact the pharmacokinetics of prucalopride when administered to young children: age; postmenstrual age (calculated age [weeks] at start of treatment plus 40 weeks gestational age); body weight; and creatinine clearance (CrCL). CrCL was calculated according to equation (1) (Schwartz et al. 1976; and further adjusted by Rowland and Tozer 2010 to accommodate body weight) (Schwartz et al. 1976; Rowland and Tozer 2010).

(1) $$\mathrm{CrCL}[\mathrm{mL}/\min]=\frac{42.5\cdot \mathrm{Height}[\mathrm{cm}]}{\mathrm{Serum}\;\mathrm{Creatinine}\;[\mu \mathrm{mol}/L]}\cdot {\left(\frac{\mathrm{Weigh}t_{i}\left[\mathrm{kg}\right]}{70}\right)}^{0.7}$$

Intercompartmental clearance (Q), CL, *V*2, and *V*3 parameters were scaled across the ages from 6 months to 18 years by body size, using standard allometric scaling equations (2) and (3), where CL_*i*_ is the clearance in the *i*th (pediatric) patient, CL_TV_ is the clearance in a reference 70 kg patient, WT_*i*_ is the weight of the *i*th (pediatric) patient, *V*_*i*_ is the volume of distribution of the *i*th (pediatric) patient, and *V*_TV_ is the volume of distribution in a 70 kg reference patient.

(2) $$CL_{i}=CL_{\mathrm{TV}}{\left(\frac{WT_{i}}{70}\right)}^{3/4}\;\mathrm{or}\;Q_{i}=Q_{\mathrm{TV}}{\left(\frac{WT_{i}}{70}\right)}^{3/4}$$

(3) $$V_{i}=V_{\mathrm{TV}}{\left(\frac{WT_{i}}{70}\right)}^{1}$$

Prucalopride is mainly excreted unchanged in urine (Shire. Resolor SmPC, Smith et al. 2012). The maturation of renal filtration rate was incorporated in the model as described by Rhodin et al. (2009) for children aged between 6 months and 2 years (equation (4)), where maturation_GFR_ is the fractional glomerular filtration rate (GFR) and PMA is postmenstrual age in weeks. It was assumed that the renal function is constant in the age range 2–20 years (after including body size as described in equation (1)), as suggested by Hogg et al. (2003), and that it declines linearly with age for individuals older than 20 years of age, as presented by Rowland and Tozer (2010). This latter function was not included in the current model as no patients older than 18 years were included in this analysis.

(4) $$\mathrm{Maturatio}n_{\mathrm{GFR}}=\frac{\mathrm{PM}A^{3.4}}{47.7^{3.4}+\mathrm{PM}A^{3.4}}$$

According to equation (4), the fractional GFR is expected to be over 97.5% for a 24‐month‐old child. Fully mature and stable renal function was therefore assumed for children older than 24 months (postnatal age) and up to 20 years of age. This maturation function was included as a covariate on clearance, resulting in the following overall equation for clearance.

(5) $$CL_{i}=CL_{\mathrm{TV}}{\left(\frac{WT_{i}}{70}\right)}^{3/4}\mathrm{Maturatio}n_{\mathrm{GFR}}\;$$

Patients with missing covariate information were either assigned the median value of that covariate in the data file, or were assigned a value based on imputation from their other covariate values. No step‐wise covariate model building was performed using the data from SPD555‐303.

### Available data

Missing observations, observations without a date or time of dosing or sampling, and observations that were below the LLOQ of the relevant assay were excluded from the analysis. In total, 13 prucalopride concentration observations from PRU‐USA‐12 were excluded owing to missing sampling times or because they were below the LLOQ. In addition, 47 prucalopride concentration observations from SPD555‐303 were excluded from the analysis: 25 records (10% of the available samples) contained concentration observations below the LLOQ; one record was labeled ‘not analyzed or no sample’; and 21 records had missing dosing information. These exclusions resulted in a final analysis dataset of 481 records from 38 of 38 patients in PRU‐USA‐12, and 244 records from 106 of 107 randomized patients in SPD555‐303.

## Results

### Patient demographics

A summary of patient demographics is shown in Table 2. In the PRU‐USA‐12 study (*n *=* *38), median age was 8.5 years (range, 4.0–12.0 years), median body weight was 27.9 kg (range, 15.0–61.0 kg), and 34.2% of patients were girls. In the SPD555‐303 study (*n *=* *137), median age was 7.9 years (range, 1.7–18.0 years), median body weight was 24.0 kg (range, 11.0–110.0 kg), and 57.7% of patients were girls.

**Table 2:** Summary of patient demographics in the PRU‐USA‐12 and SPD555‐303 studies

	PRU‐USA‐12					SPD555‐303				
Parameter	*n*	Mean	Median	Min	Max	*n*	Mean	Median	Min	Max
Calculated age, years	38	8.2	8.5	4.0	12.0	137	8.3	7.9	1.7	18.0
Postmenstrual age, years	38	8.9	9.3	4.8	2.8	137	9.1	8.6	2.4	8.7
Body weight, kg	38	30.0	27.9	15.0	61.0	137	32.4	24.0	11.0	110.0
CrCL, mL^ ^min^−1^	38	82.8	78.2	46.1	161	137	71.9	64.8	27.9	180.0
Sex, female, *n* (%)	13 (34.2)					79 (57.7)				

Postmenstrual age was the calculated age (weeks) at start of treatment plus 40 weeks gestational age.

CrCL, creatinine clearance.

### Population pharmacokinetic model validation in pediatric patients

**Figure 2:**
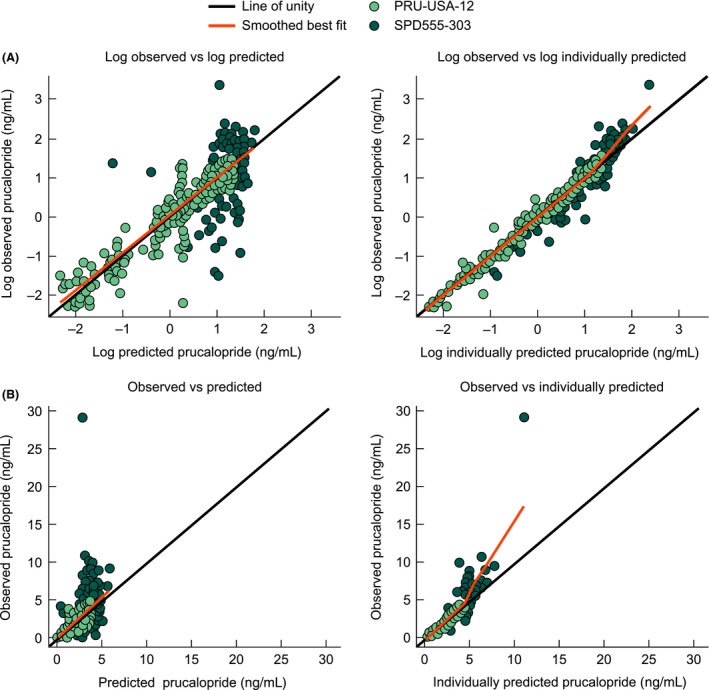
Predicted and individually predicted plasma prucalopride concentrations versus observed plasma prucalopride concentrations for (A) log‐transformed and (B) untransformed data.

**Figure 3:**
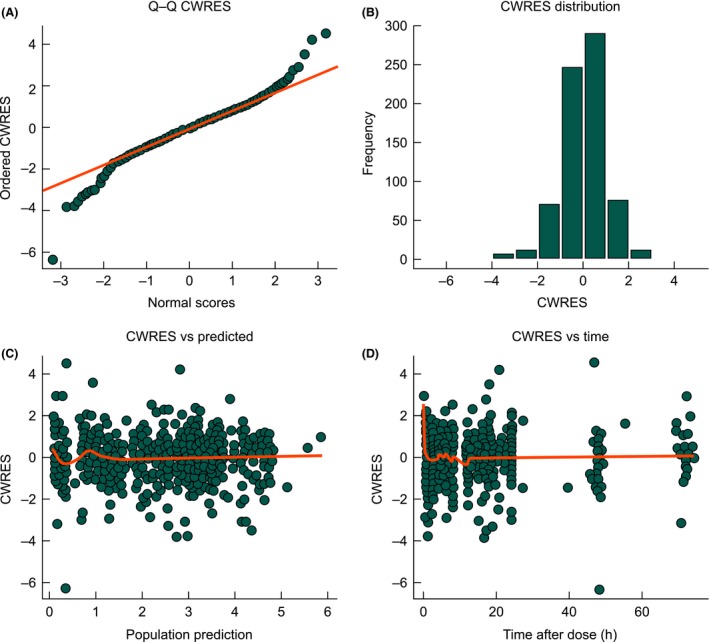
(A) Q–Q plot of ordered conditional weighted residuals versus normal scores, (B) frequency histogram of the conditional weighted residuals, (C) conditional weighted residuals versus population predictions, and (D) conditional weighted residuals versus time after dose. Orange lines are smoothing functions indicating the trends in the data. CWRES, conditional weighted residuals.

The parameter estimates for the population PK model are presented in Table 3. Goodness‐of‐fit plots demonstrated a good correlation between predicted and observed data across both studies (Figs 2 and 3).

**Table 3:** Prucalopride parameter estimates for the model using data from the PRU‐USA‐12 and SPD555‐303 studies

Parameter	Estimate	Relative SE (% CV)	95% CI
Structural model			
CL_PRU‐USA‐12_ (l h^−1^)^a^	22.9	2.4	21.9–24
CL_SPD555‐303_ (l h^−1^)^a^	20.1	3.6	18.6–21.5
*V*2 (l)^a^	446	3.3	417–475
Q (l h^−1^)^a^	16.9	15	11.7–22
*V*3 (l)^a^	248	7.9	210–286
*K*_*a*_1 (h^−1^)	0.792 fixed	–	–
*K*_*a*_2 (h^−1^)	3.87 fixed	–	–
MTIME (h)	0.734 fixed	–	–
*F*1	0.858 fixed	–	–

Parameter	IIV estimate (% CV)	Relative SE (% CV)	*η*‐shrinkage (%)
Statistical model			
CL_PRU‐USA‐12_	0.0151 (12%)	28	6
CL_SPD555‐303_	0.1191 (35%)	33	24
*V*2	0.0202 (14%)	47	58
*V*3	0.158 (40%)	68	70
*K*_*a*_1	0.794 fixed	–	–
*K*_*a*_2	0.507 fixed	–	–
Residual error PRU‐USA‐12	0.142 (14%)	9.8	–
Residual error SPD555‐303	0.35 (35%)	16	–

a Scaled to a body weight of 70 kg.

CI, confidence interval; CL, clearance; CV, coefficient of variation; *F*1, bioavailability; IIV, interindividual variation; *K*_*a*_1, absorption rate at time less than MTIME; *K*_*a*_2: absorption rate at time greater than MTIME; MTIME, cut‐off time between first and second absorption rate; Q, intercompartmental clearance; SE, standard error; *V*2, volume of distribution of the central compartment; *V*3: volume of distribution of the peripheral compartment.

Plots of post hoc random‐effect parameter distributions are shown in Figure S1. Random‐effect parameters (*η*) for CL were centered around 0 (Table 3); *η*‐shrinkage for CL was 6% for PRU‐USA‐12 and 24% for SPD555‐303, but *η*‐shrinkage for *V*2 and *V*3 was large at 58% and 70%, respectively. This shows that the sparse data from SPD555‐303 did not contain sufficient information to support the estimation of these parameters. Overall, *ε*‐shrinkage in the final model was 19%.

**Figure 4:**
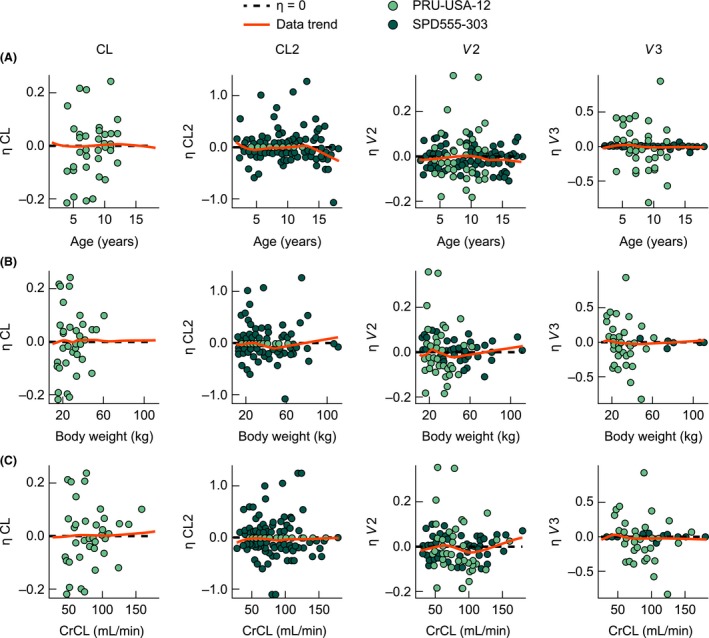
Random‐effect estimates (*η*) for clearance in PRU‐USA‐12 (CL) and SPD555‐303 (CL2), volume of distribution of the central compartment (*V*2), and volume of distribution of the peripheral compartment (*V*3) plotted against (A) calculated age, (B) body weight, and (C) creatinine clearance. CL, clearance in PRU‐USA‐12; CL2, clearance in SPD555‐303; CrCL, creatinine clearance; *V*2, volume of distribution of the central compartment; *V*3, volume of distribution of the peripheral compartment.

Taking weight into account in the model resulted in a strong correlation of CL, *V*2, and *V*3 with age, body weight, and CrCL (Fig. S2). Random‐effects estimates for CL, *V*2, and *V*3 plotted against age, body weight, and CrCL are shown in Figure 4 for both PRU‐USA‐12 and SPD555‐303. The data indicate that the allometric relationships between CL, *V*2, and *V*3 and body weight adequately accounted for body size and age in this pediatric population.

Summary statistics of individual post hoc parameter estimates for patients in PRU‐USA‐12 (receiving prucalopride 0.03 mg kg^−1^ up to a maximum of 2 mg) and SPD555‐303 (receiving prucalopride 0.04 mg kg^−1^ up to a maximum of 2 mg) are shown in Table 4. Mean (range) area under the plasma concentration–time curve (AUC) at steady state was 62.3 (40.5–82.7) ng mL^−1^ h in PRU‐USA‐12 and 100.3 (22.7–286.0) ng mL^−1^ h in SPD555‐303.

**Table 4:** Mean post hoc parameter estimates from individual empirical Bayes analysis for the PRU‐USA‐12 and SPD555‐303 studies

	PRU‐USA‐12			SPD555‐303		
	(*n *=* *38; dose = 0.03 mg kg^−1^)			(*n *=* *137; dose = 0.04 mg kg^−1^)		
Parameter	Mean	Minimum	Maximum	Mean	Minimum	Maximum
CL (l h^−1^)	12.1	3.13	22.9	11.7	4.48	75.6
*V*2 (l)	192	87.9	399	207	68.2	721
AUC (ng mL^−1^ h)	62.3	40.5	82.7	100.3	22.7	286.0
*C*_ss_ (ng mL^−1^)	–	–	–	4.18	0.945	11.9
*C*_0 h_ (ng mL^−1^)	–	–	–	2.64	0.275	10.5

AUC, area under the plasma concentration–time curve from time zero to infinity; CL, plasma clearance; CrCL: creatinine clearance; *C*_ss_, steady‐state plasma concentration; *C*_0 h_, steady‐state predose plasma concentration; *V*2, volume of distribution of the central compartment.

### Simulated plasma concentration–time profiles for prucalopride in pediatric patients

**Figure 5:**
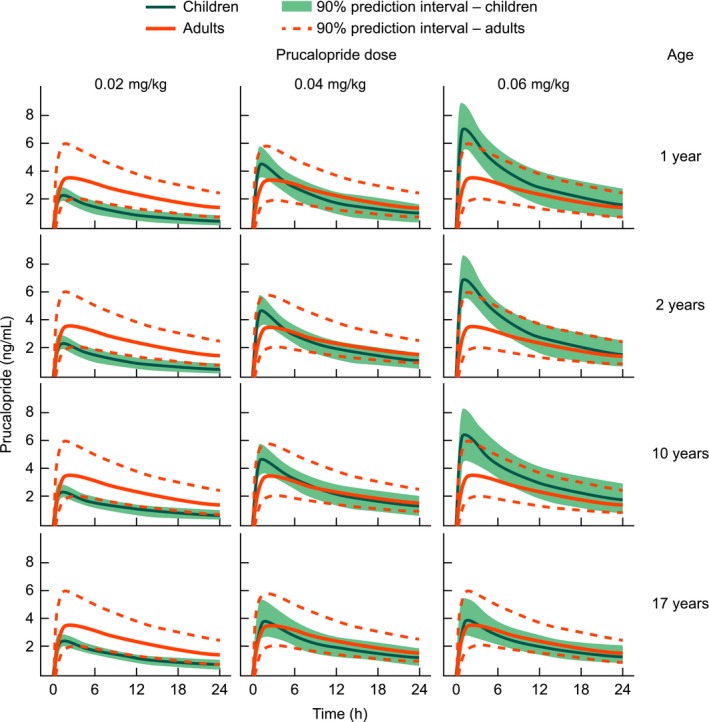
Simulated single‐dose plasma concentration–time profiles for prucalopride 0.02, 0.04, or 0.06 mg kg^−1^ in patients aged 1–17 years. The orange solid line represents the expected median plasma concentration in adults after a single 2 mg dose of prucalopride.

The model was used to simulate single‐dose and steady‐state plasma concentration–time profiles for patients 1–17 years when treated with a once‐daily dose of prucalopride 0.02 mg kg^−1^, 0.04 mg kg^−1^, or 0.06 mg kg^−1^ (with a maximum dose of 2 mg). Plots of simulated profiles for children following treatment with a single administration of each of these doses compared with simulated adult profiles are shown in Figure 5. For all ages, a single dose of prucalopride 0.02 mg^ ^kg^−1^ was predicted to result in *C*_max_ values at the lower end of those expected in adults, whereas the 0.04 mg kg^−1^ dose was expected to reach *C*_max_ within the range of that expected in adults for all age ranges. In younger children, the 0.06 mg kg^−1^ dose was expected to have a *C*_max_ higher than that of a 2 mg dose in adults. In 17 year olds, this dose resulted in a *C*_max_ similar to that observed in adults receiving a 2 mg dose.

**Figure 6:**
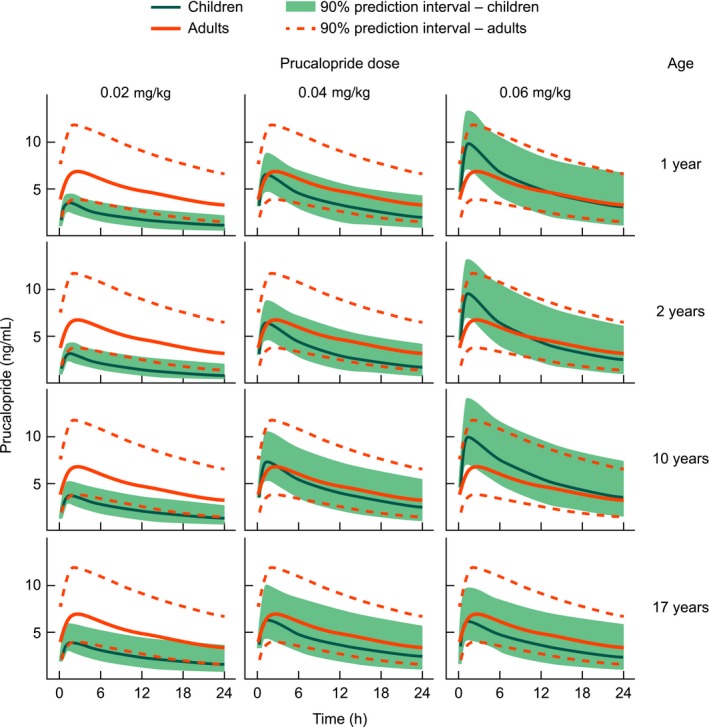
Predicted steady‐state plasma concentration–time profiles following administration of prucalopride 0.02, 0.04, or 0.06 mg kg^−1^ once daily to patients aged 1–17 years. The orange solid line represents the expected median plasma concentration at steady state in adults receiving a once‐daily 2 mg dose of prucalopride.

Simulated prucalopride plasma concentration–time profiles at steady state in patients aged 1–17 years after a once‐daily dose of prucalopride 0.02, 0.04, or 0.06 mg kg^−1^ were compared with profiles in adults taking 2 mg once daily (Fig. 6). Across the entire age range, a once‐daily dose of prucalopride 0.04 mg kg^−1^, with a maximal dose of 2 mg, was predicted to result in steady‐state *C*_max_ values close to those observed in adults after a 2 mg once‐daily dosing regimen.

It should be noted that model simulations were performed with clearance based on SPD555‐303 and absorption rate constants for the oral solution of prucalopride. In SPD555‐303, the oral solution was used for doses of up to 2 mg, but the maximal 2 mg dose was generally administered as a tablet. Adapting the model to include the rate of tablet absorption based on adult PK data did not substantially change the results of the simulations (data not shown).

## Discussion

PK studies in children are of great importance because many drugs used in pediatric clinical practice lack an evidence‐based dosing regimen and are prescribed in an off‐label or unlicensed manner (Kimland and Odlind 2012). Off‐label dosing in children is usually extrapolated from the adult dose and scaled to body weight in a linear manner; however, child development is marked by nonlinear changes in body composition and variable rates of maturation of enzyme pathways and renal function (Kearns et al. 2003; Johnson 2008). Thus, empirical, body weight–based dosing can lead to over‐ or underdosing in children, thereby producing toxicity or reduced efficacy. In order to correctly account for the developmental changes in physiology that occur during childhood, it is necessary to conduct PK studies in a wide age range, from infants to adolescents (Ince et al. 2009; Anderson and Holford 2013).

The population approach has facilitated PK studies in children because it enables the analysis of sparse‐sampling datasets, as well as datasets derived from clinical studies in which different doses were used (De Cock et al. 2011; Knibbe and Danhof 2011; Knibbe et al. 2011). In addition, by taking a small number of blood samples and/or a small volume of blood per individual from a sample of children, population PK studies fulfil the requirements for establishing evidence‐based dosing regimens while addressing the ethical and safety concerns of parents, pediatricians, and regulatory bodies regarding the conduct of PK studies in children (Meibohm et al. 2005; Anderson et al. 2006).

The initial pediatric study of prucalopride (PRU‐USA‐12) (Winter et al. 2013) evaluated the effects of prucalopride 0.03 mg kg^−1^ (approximately equivalent to a 2 mg dose in an adult weighing 70 kg) in children aged 4–12 years with functional constipation. In that study, full PK sampling was performed following the first dose of prucalopride, and the traditional noncompartmental analysis showed that the mean prucalopride *C*_max_ in children was approximately 15% lower and the mean prucalopride AUC in children was 30–40% lower than in adults. In the SPD555‐303 pediatric study (Mugie et al. 2014), the dose of prucalopride was increased to 0.04 mg kg^−1^, up to a maximum of 2 mg, in an attempt to produce a similar *C*_max_ to that observed in adults, while acknowledging that pediatric patients would still be exposed to a lower prucalopride AUC than adults. To match the prucalopride AUC in pediatric patients to that observed in adults, the prucalopride dose would have needed to be increased further, which would have exposed the pediatric patients to a higher *C*_max_ than that in adults, an outcome that was not desirable because of potential toxicity concerns.

This study has shown that the initial pediatric model could not completely describe the observed sparse prucalopride concentration data in SPD555‐303. Goodness‐of‐fit plots indicated a bias in the population predictions, suggesting underprediction of prucalopride concentrations. Furthermore, the initial model, based on richly sampled data, did not account for the large interpatient variability observed in SPD555‐303. Combining the sparsely sampled data from SPD555‐303 with the single‐dose data from PRU‐USA‐12 provided an improvement in the model fit as judged by a considerable drop in objective function. Furthermore, the residual error (*ε*) in SPD555‐303 was much larger than that in PRU‐USA‐12 (35% in SPD555‐303 vs. 14% in PRU‐USA‐12). The increased residual error in SPD555‐303 reflects the sparse PK sampling in this study (one sample collected near *C*_max_ on day 1, and two samples collected near *C*_min_ at weeks 8 and 24 from each patient) compared with full PK sampling from the well‐controlled, single‐dose study PRU‐USA‐12. The sparse PK sampling from SPD555‐303 made it more difficult to distinguish among interpatient variability, interoccasion variability, or residual variability than in PRU‐USA‐12. A slightly lower typical value for CL in SPD555‐303 appeared to provide a better description of the trial data. The value for CL was only 12% lower than in PRU‐USA‐12, but in combination with a considerably larger interindividual variability in CL, it provided a better description of the observed prucalopride concentrations. For future simulations or for linking of exposure to pharmacodynamics measurements, it is important to take this variability into account. The other estimated parameters were in line with the results obtained in the previous analysis. The slightly lower apparent value for clearance in SPD555‐303 likely does not reflect a true mechanistic difference in clearance between the study populations, but is more likely the result of differences in trial design, single‐ versus multiple‐dose sampling, compliance in the outpatient clinical setting of SPD555‐303, or differences in relative bioavailability of the formulations used.

The final model provided an adequate description of the PK of prucalopride as judged by the goodness‐of‐fit plots. The functional allometric scaling of the disposition parameters was suitable to account for body size–related differences among children of different ages. No additional correlations were observed between random‐effect parameters (*η*) for CL and *V* and continuous covariates such as age, weight, and CrCL. Estimates of exposure (AUC) based on the final model were in agreement with previous simulations based on an adult PK model (data on file). *η*‐shrinkage of CL, the main PK parameter determining steady‐state exposure, was 6% for PRU‐USA‐12 and 24% for SPD555‐303. *ε*‐shrinkage in the final model was 19%. *η*‐ and *ε*‐shrinkage values of less than 20–30% indicate that individual parameter estimates are reasonably accurate (Karlsson and Savic 2007; Savic and Karlsson 2009).

The primary objectives in developing the model were to provide a good description of the PK of prucalopride in children and to assess whether the 0.04 mg kg^−1^ dose in SPD555‐303 produced a prucalopride *C*_max_ and exposure in children that was similar to the *C*_max_ and exposure in adults. The simulations based on the final population PK model indicated that a once‐daily dose of prucalopride 0.04 mg kg^−1^ resulted in similar steady‐state *C*_max_ concentrations, but slightly lower AUC values than those observed in adults after prucalopride 2 mg once daily, which matched the a priori expectations based on the PK data in children receiving 0.03 mg kg^−1^ (Winter et al. 2013). The mean predicted AUC based on the final model was 100.3 ng mL h^−1^, slightly lower than the adult AUC of 109.3 ng mL h^−1^ (data on file). Overall this suggests that the observed lack of efficacy in study SPD555‐303 was not caused by insufficient prucalopride exposure in the pediatric patients in the study.

A possible explanation for the negative results of the SPD555‐303 study is the differences in the characteristics of constipation among children, adolescents, and adults. For example, in the majority of children with the condition, voluntary withholding of feces caused by a fear of painful defecation plays an important role in functional constipation; however, this is very rarely involved in the onset or persistence of constipation in adults (Solzi and Di Lorenzo 1999). Approximately 60% of patients in the SPD555‐303 study had a history of excessive volitional stool retention so it seems likely that this may have had an impact on the results. Additional factors that underlie the differences between chronic constipation in children versus adults may have also contributed to the negative results of the SPD555‐303 trial,

## Conclusions

This population PK analysis indicates that prucalopride 0.04 mg kg^−1^ up to a maximum of 2 mg once daily in pediatric patients in the SPD555‐303 phase 3 trial produced a similar prucalopride *C*_max_ and a reduction of approximately 10% in AUC (Mugie et al. 2014) compared with adult patients. The results of our analysis indicate that the negative efficacy results of the SPD555‐303 trial cannot be explained by differences in prucalopride PK exposure in children compared with adults.

## Author Contributions

EvS and MM were the primary authors involved in model development. All authors contributed to the analysis and interpretation of the data, and to the drafting of the manuscript.

## Disclosures

All authors have completed the Unified Competing Interest form at http://www.icmje.org/coi_disclosure.pdf and declare that: EvS is an employee of SGS Exprimo NV, Mechelen, Belgium. MB is an employee of Emma Children's Hospital/Academic Medical Center, Amsterdam, Netherlands. ST is an employee of Shire, Wayne, PA, USA, and holds stock/share options in Shire. AL was an employee of Shire at the time the study was conducted and holds stock/share options in Shire. MM is an employee of Pharmetheus AB, Uppsala, Sweden, and is a consultant for AstraZeneca, Danone, Johnson & Johnson, Sucampo, and Zeria.

## Supporting information


**Figure S1.** The pediatric population PK model: post hoc random‐effect parameter (*η*) distributions for CL (clearance in PRU‐USA‐12), CL2 (clearance in SPD555‐303), and V2 (volume of distribution of the central compartment). Orange lines show the observed mean and density and blue lines show the predicted mean and density. *Ε* shrinkage in the final model was 19%. CL, clearance in PRU‐USA‐12; CL2, clearance in SPD555‐303; *V*2, volume of distribution of the central compartment.

**Figure S2.** The pediatric population PK model: relationships between clearance (CL), volume of distribution of the central compartment (*V*2), volume of distribution of the peripheral compartment (*V*3), and (A) calculated age, (B) body weight, and (C) creatinine clearance. CL, clearance; CrCL, creatinine clearance; *V*2, volume of distribution of the central compartment; *V*3, volume of distribution of the peripheral compartment.
